# Selecting the Right Toys for Your child is Not a Child’s Play

**DOI:** 10.4103/0970-0218.66867

**Published:** 2010-04

**Authors:** Harshal T Pandve

**Affiliations:** Department of Community Medicine, Smt. Kashibai Navale Medical College, Narhe, Pune - 411 041, India.E-mail: dr_harshalpandve@yahoo.co.in

Sir,

Toys are essential part of child’s early years of life. Toys serve multiple purposes in child’s development. Toys not only provide entertainment but also fulfill some educational role. A toy enhances observational capacity and stimulates creativity. They play major role in development of physical as well as mental skills which are necessary in later life.

Today the rising issue is “whether the toys are safe enough?” It is little known that toys which give immense joy to children could also provide grief to them as well as to their parents. Toys could potentially be dangerous to their health or life threatening for several reasons. We all know babies put every single thing they get in their little hands into their mouth and that include the lovely, bright and colorful plastic toys we buy. But research has now shown that those very small plastic toys could be poisoning your baby every single time they put it into their mouth.(1)

A campaign launched by Generation Green in the late 1990s focused on phthalates and their risks to kids. Phthalates are chemicals used to soften PVC plastic. They could be ingested by children when sucking on toys or baby products made with polyvinyl chloride (PVC). Ingestion of phthalates has been linked to increased risks of cancer, kidney damage and interference with reproductive development. (2) In India the study conducted by Toxic Link, an environmental NGO, showed that dangerous levels of lead and cadmium were found in PVC soft toys collected from wholesale markets of Mumbai, one of the largest manufacturing and supply centres for unbranded toys. PVC is a synthetic resin used as the basic material in plastics, among other things. The study indicates that children are being exposed to severe health hazards caused by these metals, ranging from liver damage to disruption in mental growth. Plastics like PVC are chemically dependant, and need additives like lead, cadmium and other chemicals to make them usable. However, these additives leach from the PVC and contaminate human bodies, putting especially children at risk. Lead and cadmium are proven poisons, being neurotoxins and nephrotoxins, respectively. Similar studies were conducted in Chennai and Delhi but there are no legal and binding standards that stipulate the safe limits of heavy metal content in toys (plastic or otherwise) that are made locally or imported. (3) A similar kind of study was done by the Consumer Unity and Trust Society as premier consumer rights organization. (4)

Many countries have passed safety standards limiting the types of toys that can be sold. Most of these seek to limit potential hazards. Children, especially very small ones, often put toys into their mouths, so the materials used to make a toy are regulated to prevent poisoning. Materials are also regulated to prevent fire hazards. Children have not yet learned to judge what is safe and what is dangerous, and parents do not always think of all possible situations, so such warnings and regulations are important on toys.(5)

In India though the Bureau of Indian Standards (BIS) has clearly formulated the standards relating to toy safety in terms of their physical form and toxicity, most of the manufacturers are either not following safety norms or are completely oblivious of the same. (4) The enforcement of guidelines is yet to be made mandatory for domestic toy manufacturers. Toys, particularly soft PVC toys, have not been investigated as one of the possible sources of harmful metals.(3) India is also a huge market for non-branded toys from other countries like China where the regulation and norms for toys manufacturing are not followed strictly and bear potential danger to the children. Government authorities need to take effective steps to regulate the manufacturing and marketing of toys as per the formulated norms.

Role of parents is most important in this issue. To protect the child from any injury or hazard from toys, parents must follow some guidelines. Parents should avoid buying non-branded toys, plastic toys, toys on internet, and brightly colored toys as they contain higher content of lead. Also, parents should carefully read the instructions given on the toys or its manual; preferably buy toys made up of cloths or wooden. If the child is having frequent health problems without any obvious reason, seek experts opinion as it can be due to the toys he is playing with, supervise your child while playing with toys to avoid any mouth contact with it. These are some simple things that can be done to avoid any injury or hazard to your child.
